# Subjective and Objective Symptomatology of Tuberculosis

**Published:** 1915-07

**Authors:** Louis C. Rouglin

**Affiliations:** 807-808 Candler Building


### SUBJECTIVE AND OBJECTIVE SYMPTOMATOLOGY OF TUBERCULOSIS.

By Louis C. Rouglin, M. D.

In discussing the symptomatology of plumonary tuberculosis, it may be well from the outset to state, that there is no group of symptoms or any one symptom, whether subjective or objective, that is pathogonomic of tuberculosis; there are no symptons in this disease, which are distinctly typical and which may not occur in other pulmonary affections, moreover, the symptoms manifested are not indications of the true pathology of the condition. Mild symptoms with grave pathological conditions are not uncomomn, while the reverse is often true. Yet it is possible within certain flexible limits to describe the symptomatology, and for this purpose it is best to sub-divide the symptons according to the mode of onset, which may be classified as

(a) Acute.
(b) Non Acute.
(c) Chronic.

The acute type may be divided into the

1. Acute miliary tuberculosis of the pneumonic type.
2. Acute septic tuberculosis.

3. Acute pneumonic phthisis.

4. Acute broncho-pneumonic phthisis.

5. Acute pleuritic phthisis.

6. Initial hemmorhagic pulmonary phthisis.

8. Pthisis of the acute rheumatic, influenced or LaGrippe type.

In all the above the symptoms are essentially the same as manifested by their respective associated diseases, and they usually have a definite relation to the underlying pathological condition.

The non-acute types of this disease may be classified according to the symptoms, as those of the

(a) Anemic type. In these cases there may be no other symptoms existing suggestive of the pulmonary trouble. In females this is usually accompanied by irregular, scanty or completely suppressed menstruation and the patient usually feels run-down, with perhaps but little loss of weight, and usually some shortness of breath. Dizziness and pallor are the most striking features.

(b) Dyspeptic type. This begins with severe dyspeptic disorders, loss of appetite, vomiting, anorexia, gastralgia with acid eructations, and followed by insomnia with marked disturbances of a psycho-neurotic nature. Such are the predominant characteristics of this type.

(c) Laryngeal type. The first complaint is that of a slowly developing hoarseness, which is usually considered of a simple catarrhal character. Complete loss of voice for several weeks, is not uncommon in these cases. There may be no cough or expectoration, or fever and there is usually no loss of strength or weight. Only laryngoscopic examination discloses evidence and reveals the true condition.

(d) Following other infectious diseases, such as Pneumonia, Typhoid Fever, Measels, etc., in which convalescence is slow and unsatisfactory and the patient fails to regain his former strength and health.

In the chronic type of this disease, the manifestations are so insidious, as to escape the attention of the patient for a long time, and it will be best to describe them in the manner and order in which they are ordinarily observed by a large majority of the patients. The individual begins to notice a slow but sure decline in strength, he tires easily and there is lack of physical energy. He is inclined to rest after slight exertion; although there may be mental energy and a desire for work, he does not feel physically able to exert himself further. Patients will often express themselves as not being able "to get themselves together." This is usually, but not invariably, followed by a slight cough which may be described as not more than a slight clearing of the throat. It is persistent and gradually gets worse. It is usually most severe, on first lying down at night, or on arising in the morning, or may be most troublesome immediately after eating. The cough is accompanied by a variable amount of expectoration, from half a dram to over a pint in twenty-four hours. The expectoration, like the cough, may be noticeable on retiring at night or on arising in the morning. In some cases it is expelled with apparent ease, while in others it is brought up only with difficulty.

Pain in the chest, while not a constant feature, is commonly met with; it may be of the acute neuralgic and transient type, but more often of the type described by patients as a "heaviness of the chest." Intercostal neuralgia is commonly complained of, with this condition insomnia followed by irritability of temperment is quite a constant accompaniment.

Increase of body temperature is perhaps one of the very earliest indications of tuberculosis. It may be only a slight increase to 99. to 100.5 F. in the evening, or it may often be as high as 101 or 102,5. F, yet the patient will be totally unconscious of his fever. A tolerance to high temperature is, in my opinion, peculiarly significant and practically characteristic of tuberculosis.

Dyspnea with more or less cyanosis, on slight exertion, is more or less complained of; it is not unusual, however, to meet a well marked case of tuberculosis boasting of being "long winded." Night sweating, or perspiration confined to localized areas, such as the head or back of the neck, is quite a noticeable symptom. Occasionally with the above we get a history of repeated attacks of "colds," fits of sneezing, and of occasional expulsion of blood stained sputa, which the patient will always declare "comes from the throat, and not from the lungs."

All of the above symptoms may go on for months, without the patient realizing that there is anything serious the matter with him. It is only when emaciation becomes marked, or when any of the above mentioned symptoms, especially the cough, or fever becomes exceedingly troublesome, that the patient may then for the first time present himself to the physician for examination.

Of the objective symptoms, there are very few of any importance, that do not justly belong under the heading of "Inspection" which will be ably discussed by the gentleman assigned to the subject. The anemia and emaciation may be very striking; the skin is usually dry and has a sallow cachectic appearance; the cheeks may be flushed, while the hands feel cold and clammy. The complexion is either muddy or extremely fair with the superficial veins prominent, and a peculiar "glistening" look (Facies Tubercular) in the eye. All other objective symptoms will come more properly under the heading of Physical Examination.

In closing I wish to state, that in order to derive any positive or definite conclusion from the symptomatology of tuberculosis, it is essential to have, (a) An intelligent patient. (b) An educated physician (c) Long experience upon which to base proper deductions and interpretations from the symptoms, and lastly (d) it must be corroborated by a thorough physical examination, and all other means at our command to make a positive diagnosis.

807-808 Candler Building.

